# Genome Wide Mapping Reveals PDE4B as an IL-2 Induced STAT5 Target Gene in Activated Human PBMCs and Lymphoid Cancer Cells

**DOI:** 10.1371/journal.pone.0057326

**Published:** 2013-02-25

**Authors:** Zsuzsanna S. Nagy, Jeremy A. Ross, Georgialina Rodriguez, Balint L. Balint, Lajos Szeles, Laszlo Nagy, Robert A. Kirken

**Affiliations:** 1 Department of Biological Sciences, University of Texas at El Paso, El Paso, Texas, United States of America; 2 Department of Biochemistry and Molecular Biology, University of Debrecen Medical and Health Science Center, Debrecen, Hungary; University of Memphis, United States of America

## Abstract

IL-2 is the primary growth factor for promoting survival and proliferation of activated T cells that occurs following engagement of the Janus Kinase (JAK)1–3/and Signal Transducer and Activator of Transcription (STAT) 5 signaling pathway. STAT5 has two isoforms: STAT5A and STAT5B (commonly referred to as STAT5) which, in T cells, play redundant roles transcribing cell cycle and survival genes. As such, inhibition of STAT5 by a variety of mechanisms can rapidly induce apoptosis in certain lymphoid tumor cells, suggesting that it and its target genes represent therapeutic targets to control certain lymphoid diseases. To search for these molecules we aligned IL-2 regulated genes detected by Affymetrix gene expression microarrays with the STAT5 cistrome identified by chip-on-ChIP analysis in an IL-2-dependent human leukemia cell line, Kit225. Select overlapping genes were then validated using qRT^2^PCR medium-throughput arrays in human PHA-activated PBMCs. Of 19 putative genes, one key regulator of T cell receptor signaling, PDE4B, was identified as a novel target, which was readily up-regulated at the protein level (3 h) in IL-2 stimulated, activated human PBMCs. Surprisingly, only purified CD8+ primary T-cells expressed PDE4B, but not CD4+ cells. Moreover, PDE4B was found to be highly expressed in CD4+ lymphoid cancer cells, which suggests that it may represent a physiological role unique to the CD8+ and lymphoid cancer cells and thus might represent a target for pharmaceutical intervention for certain lymphoid diseases.

## Introduction

The mammalian Signal Transducer and Activator of Transcription (STAT) family is composed of 7 members (1–4, 5a, 5b and 6). STAT molecules exert critical roles in cell proliferation, differentiation and survival (reviewed in [1]). Originally, STATs were believed to be latent factors residing in the cytosol and only activated when cytokines bind to their cognate receptor after activation of Janus tyrosine kinases (JAK). Indeed the central model suggests that JAKs phosphorylate tyrosine residues on the receptor serving as docking sites for SH2 domain-containing signaling molecules such as STATs. Following docking via phosphotyrosine-SH2 interactions, STATs themselves become tyrosine phosphorylated by JAKs, disengage from the receptor and form dimers via reciprocal phosphotyrosine-SH2 domain interactions. The STAT dimer is then translocated to the nucleus to initiate gene transcription [2]–[3]. However we now know that STATS can dimerize and form tetramers in the absence of tyrosine phosphorylation and be nuclear localized to control gene regulation in many unique ways that are less understood [4]–[5].

What is clear is that STATs 1, 3, 5A and 5B are widely utilized by various cytokines and are important for the regulation of cellular growth, proliferation and death, while STATs 2, 4 and 6 promote viral defense and Th1 versus Th2 differentiation, respectively. Moreover, STAT3 and STAT5 have been found to be closely related and relevant to tumorigenesis (reviewed in [1]). Indeed, constitutively tyrosine phosphorylated forms of STAT5 are readily observed in a variety of cancerous cells and tissues of distinct origins as a result of chromosomal translocation, deregulated tyrosine kinases and viral transformation (reviewed in [1]).

The physiological role of STAT5A and B is largely derived from *in vivo* studies of STAT5A and B knockout animals. From these studies it appears clear that STAT5A is mainly involved in mammary gland development [6] and STAT5B is responsible for regulating growth via growth hormone signaling [7]. In T lymphocytes, however, they have compensatory functions: studies from STAT5A/B double deficient mice showed that they have redundant roles in mediating cell cycle progression of activated T cells [8], [9]. In addition, studies employing STAT5A/B null mice indicate that these molecules are important for lymphoid organ development as a deficiency in these proteins can result in severe combined immunodeficiency phenotype [10]. Moreover, STAT5 appears to act as a critical survival factor for T-cells, since constitutively active (i.e. tyrosine phosphorylated) STAT5 is often present in lymphoid and leukemic cancer cells among other types of tumors as compared to normal, non-transformed cells (reviewed in [1]). Furthermore, blocking STAT5 expression in human peripheral blood mononuclear cells as well as lymphoid and leukemic cancer cells severely compromise cell viability and induce apoptotic cell death [11]. Evidence from many groups suggests that STAT3 plays a similar oncogenic role to STAT5 and dependent upon the cell type, one may be more dominant [12]. New findings for this family of proteins also suggest that their cell survival promoting characteristics when un-phosphorylated can play a gene regulatory role as well [4]. These data help provide intriguing new models that suggest that pharmacological uncoupling of activated as well as un-activated STAT5 may be required to disrupt their target genes to induce cancer cell death. Thus, identifying cell survival and tumor relevant STAT5 target genes is an important goal for the development of novel anti-cancer therapies.

One method that has proven successful in identifying novel target genes is chromatin immunoprecipitation which can reveal direct transcription factor- DNA interactions [13] and allows for the identification of unknown transcription factor binding sites in novel target genes by generating a genome-wide library that can be (i) sequenced and located in the human genome, or (ii) hybridized to microarrays representing non-coding regions of the genome. Genome-wide mapping of cytokine induced STAT5 target genes have been performed and published in various cell types (T-, pre-B-, human NK-like tumor and breast cancer cells) using ChIP-clone or ChIP-Seq technologies [4] [14]–[18]. In particular, mapping IL-2 inducible STAT5 binding events and transcriptional changes in primary T cells using ChIP-Seq, gene expression microarrays, or RNA-Seq technology have been performed and published for both mouse [5], [16], [19], [20] and human [16], [19] models. These important studies primarily focused on the role of STAT5 in T helper cell differentiation and physiological immune responses.

The present work sought to identify key cell survival and tumor relevant STAT5 target genes in IL-2 dependent human leukemia cells using gene expression and promoter microarray studies. The results of which were aligned and a select pool of candidate targets were then validated in normal human activated PBMCs. Phosphodiesterase (PDE) 4B, a regulator of cyclic AMP signaling in T cells was shown to be IL-2 regulated in primed human PBMCs and CD8+ purified T cells. Interestingly this molecule was strongly over-expressed in lymphoid tumor but not primed primary CD4+ T cells.

## Results and Discussion

### A Genome-wide Approach to Identify STAT5 Specific IL-2-mediated Genes

STAT5 is a potent regulator of T cell survival as its depletion results in massive cell death of activated T and lymphoid tumor cells [11]–[12]. To date, genome-wide studies have been utilized to identify and locate IL-2 regulated STAT5 binding events and target genes in normal mouse [5], [16], [19], [20] and human T cells [16], [19] reviewed in [21]. However, in the present study we sought to identify genome-wide IL-2 mediated STAT5 gene targets from lymphoid tumor cells (Figure S1). To systematically map STAT5 binding sites on the genomic scale that we define as the STAT5 cistrome [22] and IL-2 mediated gene expression changes we employed microarrays. For these studies we first determined the STAT5 cistrome using chip-on-ChIP in IL-2 dependent Kit225 cells that were either left un-stimulated or stimulated with IL-2 for 30 minutes, followed by subsequent gene expression analysis to detect IL-2 mediated mRNA changes at 3 hours. The resultant data pools were then statistically analyzed and aligned and a select set of genes that harbored STAT5 binding sites within their promoter regions were validated using medium throughput qRT^2^PCR arrays.

### Generating a Library of IL-2 Regulated STAT5 Binding Sites

**Figure 1:**
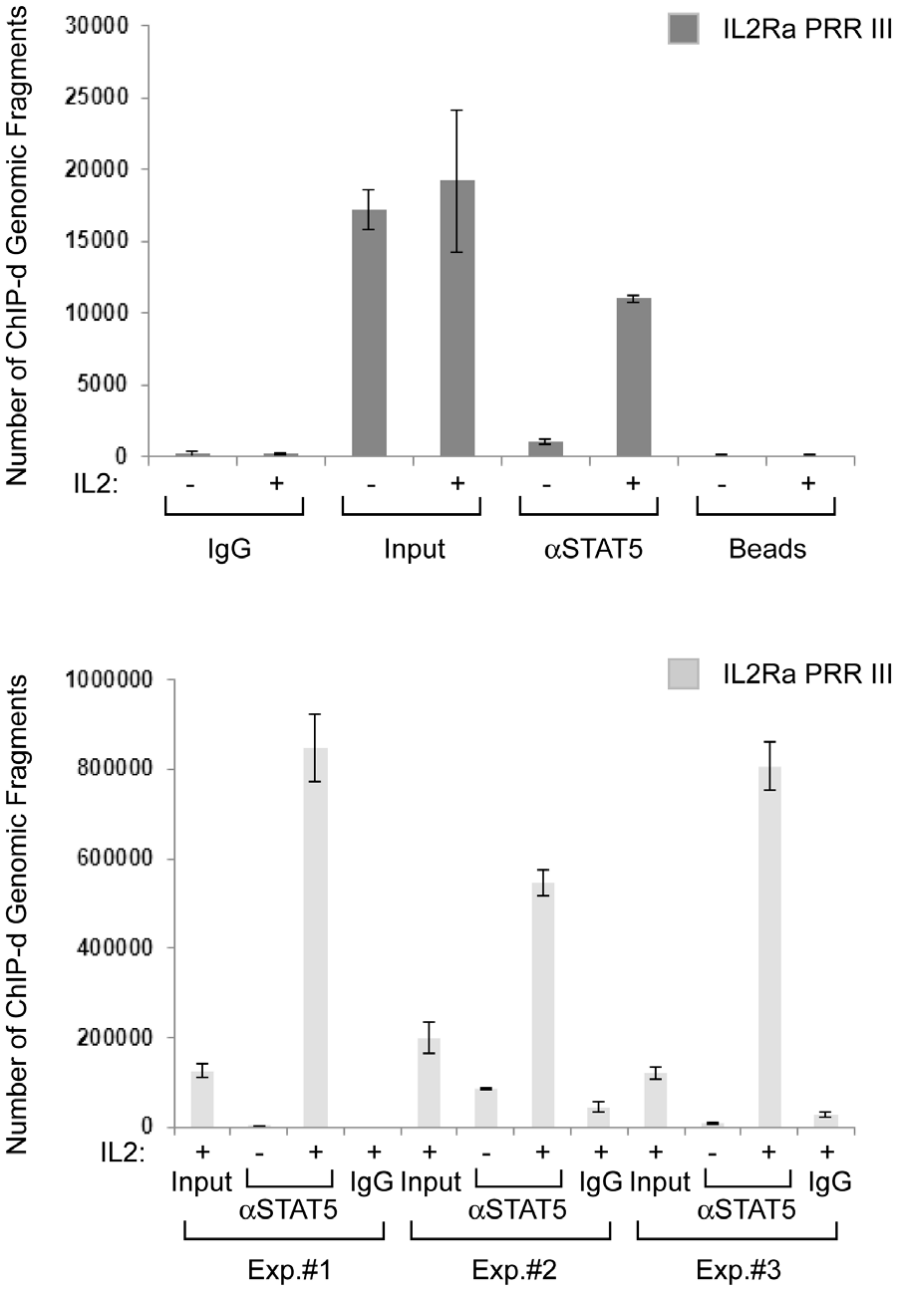
IL-2 mediated enrichment of the STAT5 binding site in the IL-2RA enhancer (A) before and (B) after amplification. (A) Kit225 IL-2-dependent human leukemia cells were made quiescent and then stimulated with medium (−) or IL-2 (+) for 30 min at 37°C, fixed with 1% formaldehyde for 10 min at room temperature and then chromatin immunoprecipitated with antibodies to C-terminal STAT5A/B mix or control IgG. The eluted DNA was amplified with primers corresponding to the human IL2RA PRR III. Representative data from three independent experiments are shown. Input material represents 5% of immunoprecipitated chromatin. Beads control represents samples in which immunoprecipitation was performed without any antibodies but otherwise was handled identically. (B) Cells were treated as described above and then for the microarray experiments the ChIP-ed DNA was randomly amplified following ligation of linkers as described in the Materials and Methods section from three independent experiments. The amplified DNA was then used as template in qPCR reactions to measure the enrichment of the IL2RA PRR III.

**Figure 2:**
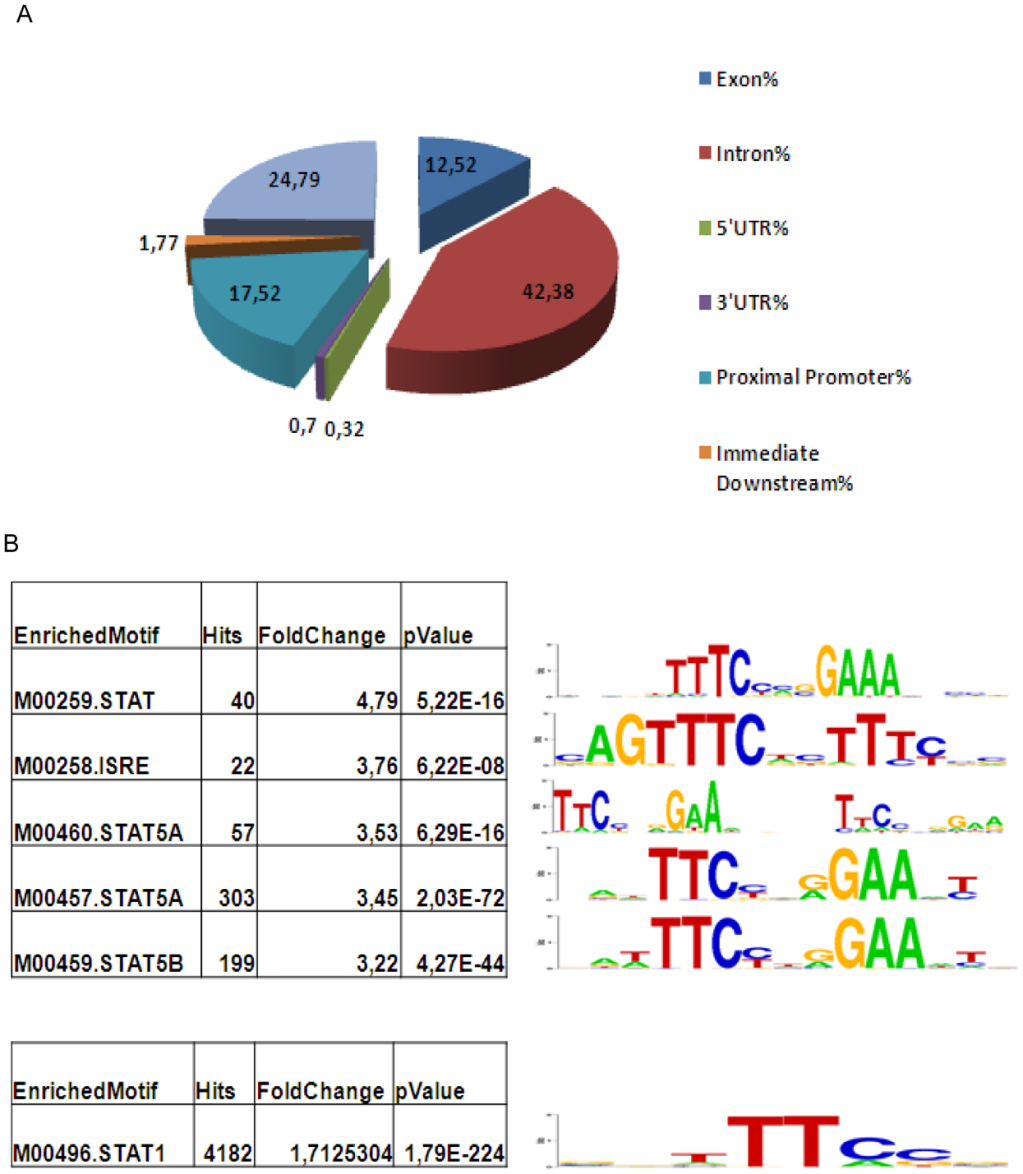
Cis-Regulatory Element Annotation System (CEAS) analysis of STAT5 binding elements. (**A**) **Genome-wide distribution of STAT5 binding sites (percent of total number of sites).** The chip-on-ChIP identified elements that fell within 300 kb from coding regions were analyzed based on their distance from nearest genes using CEAS. The pie chart represents “%” distribution. (**B**) **Enriched transcription factor binding motifs with highest fold-change (upper panel) and highest significance (lower panel).** Enriched TF binding sites and their matrices within the chip-on-ChIP identified, CEAS analyzed regulatory elements are shown.

First, three biological replicates of ChIP were performed utilizing a mixture of antibodies directed toward the C-terminal of STAT5A and STAT5B in Kit225 cells stimulated with IL-2 for 30 min. The assay was validated by measuring the IL-2 induced enrichment of the IL2RA enhancer [23] element named Positive Regulatory Region (PRR) III (Figure 1A) via STAT5 antibodies as compared to the control serum. Next, the captured DNA was amplified as described in the Materials and Methods using a random amplification protocol. To confirm that the libraries remained enriched for the positive control PRR III element post-amplification, PRR III was measured using qPCR (Figure 1B) from all the replicate experiments. Next, microarray analysis using Affymetrix GeneChip Human Promoter 1.0R arrays was carried out employing input genomic DNA (as a control) and the IL-2 stimulated samples in three biological replicates as described above. The resulting “.cel files” were then analyzed using MAT (Model-based Analysis of Tiling array, [24]) that yielded “.bed files” with the chromosomal coordinates of all ChIP-regions including p-values, MAT scores (to allow enrichment in different regions of different length to be compared directly), FDR (false discovery rate) calculations and repeat flags. Repetitive and segmental duplication elements were removed and the remaining 1581 hits were mapped with CEAS (Cis-regulatory Element Annotation System) [25] within 300 kb of annotated genomic regions. As shown in Figure 2A only about 17% of candidate genes lie within nearby promoters, while about 25% and 42% are located in enhancers and introns, respectively. These findings are in good agreement with others’ data indicating that the majority of the TF binding sites are found in intronic and intergenic regions [26]. To compare our results with previously published ChIP-Seq data sets, STAT5A and STAT5B binding sites derived from primary human CD4+ T-cells were re-analyzed (GSE27158, [16] using a ChIP-Seq analysis pipeline by Barta et al, [27]) and the resultant peaks subjected to CEAS analysis. The resulting outcomes for STAT5A and STAT5B sites distribution were as follows: promoter-bound 21% and 19%, intronic 34% and 35%, enhancer-bound 38% and 39%, respectively, indicating that the genomic STAT5 binding pattern from the current chip-on-ChIP data sets closely match the ChIP-Seq results by Liao and colleagues [16]. Interestingly only about 20% of the STAT5 binding sites were confined to promoters. Next, the enrichment of TF binding motives were also analyzed based on the CEAS assigned fold change value which represents significantly enriched motives with >2 fold-change within the ChIP regions over the whole genome. Figure 2B upper table and panel shows that the TF motives with the highest fold changes include the classical STAT binding motives TTCNNNGAA in various forms. The Interferon Sensitive Response Element (ISRE) was also significantly enriched which suggests that in certain cells STAT5 can also possibly bind to these motifs: either directly or by the assistance of another TF that binds ISRE. Figure 2B lower table and panel represent the most significantly enriched TF binding sites. Intriguingly these are TTC STAT half-sites. Perhaps in these types of cells STAT5 can bind half-sites directly or indirectly, suggesting an abnormal regulation as this type of DNA binding has not been observed *in vitro* [28].

### Identifying IL-2 Regulated Genes in Kit225 Cells Using Gene Expression Analysis

In order to identify IL-2 mediated genes in Kit225 cells, cells depleted of IL-2 were treated with IL-2 or control vehicle for 3 hours and subjected to gene expression microarray analysis in two biological replicates. From this analysis, 469 genes changed greater than 2-fold, with 129 down- and 340 up-regulated genes including several IL-2 mediated targets such as CISH, SOCS1, OSM, PIM-1, BCL6 and BCL2. This gene list was analyzed with the Ingenuity Pathway Analysis web-based software which further confirmed STAT5A and STAT5B activation status following IL-2 treatment (Figure S2) and identified cellular networks relevant to immune function including Cellular Development & Cell Cycle, Hematological System Development & Function, Reproductive System Development & Function as well as Cellular Movement, Growth & Proliferation significantly overrepresented (Figure S3).

### Identifying IL-2 Regulated Genes of the STAT5 Cistrome

**Figure 3:**
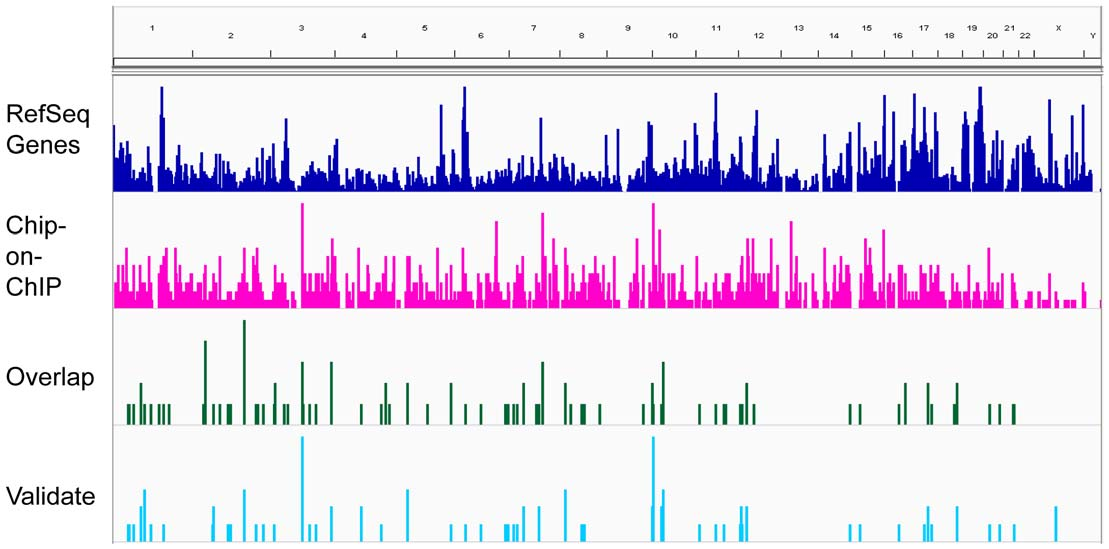
Genome-wide visualization of chip-on-ChIP identified (magenta), Gene Expression Analysis (GEA) and chip-on-ChIP overlap (green) and select (blue) putative STAT5 binding sites chosen for validation. Visualization of the results obtained from the genome-wide identification of IL-2 induced genes and STAT5 binding sites (as described in Fig. 1) using the IGV.

**Figure 4:**
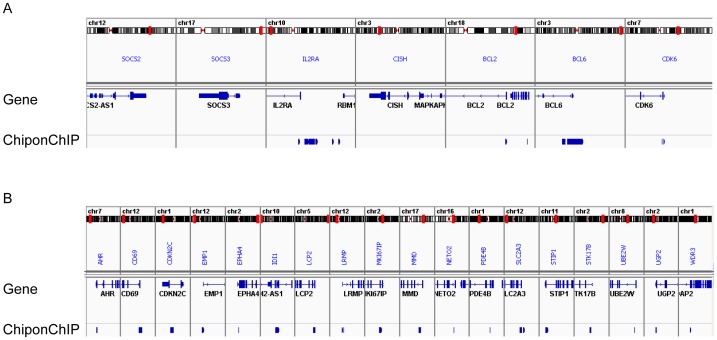
Genomic location of IL-2 regulated STAT5 binding sites. (**A**) Shown are known STAT5 target genes including SOCS2, SOCS3, CISH and those also identified by chip-on-ChIP (IL2RA, BCL2, BCL6 and CDK6) and (**B**) 18 newly identified promoter located genes visualized by the IGV using hg19.

Our aim was to discover a pool of IL-2 responsive genes that contain STAT5 regulatory sites. Therefore, we created intersect of the STAT5 cistrome and the IL-2 responsive genes using the UCSC Table Browser. First, the Affymetrix IDs of the gene expression pool were converted to genomic locations (.bed files) that were then aligned with the chip-on-ChIP results. These pools were visualized on the genomic scale using the “.bed files” and the Integrative Genomics Viewer (IGV, Figure 3). From the intersect pool comprised of 106 genes, a list of 57 genes that contained the STAT5 regulatory sites within their proximal promoter (30 genes), immediate downstream segments of the gene (7 genes), enhancer (14 genes) or first exon (6 genes) were chosen for validation in human primed PBMCs (Figure 3 and Table S1.) at the mRNA level using medium throughput qRT^2^PCR gene expression arrays (described in the Materials and Methods and statistically significant results (p-value <0.05) shown in Table 1). The known IL-2 target genes (indicated by asterisks in Table 1) BCL2, BCL6, CDK6 and IL2RA were identified as IL-2 inducible genes with STAT5 binding sites. Other known IL-2 target genes such as CISH, IFNG and FOXP3 were also identified by GEA and therefore were included as positive controls on the arrays. Although these genes are known to be regulated by STAT5, in Kit225 cells their STAT5 binding sites were not identified by the chip-on-ChIP analysis, for which we cannot rule out a cell type specific effect. To clarify these findings, IGV was used to visualize the genomic locations of known (SOCS2, SOCS3, IL2RA, CISH, BCL2, BCL6 and CDK6 (Figure 4A) underlined are those identified in our screen) and 18 unknown IL-2/STAT5 target genes (Figure 4B). Among these for instance, CD69 (up 2.3-fold) has been shown to influence Th17 differentiation, which is a known STAT5-dependent process [29]. CDKN2C, otherwise called as p18(INK)4c, is a known inhibitor of G1 cell cycle initiation, which here is down-regulated about 2-fold by IL-2. Lymphocyte Cytosolic Protein (LCP) 2 (1.7-fold up) is a target for Zap70 kinase in T lymphocytes and its deficiency is known to induce an absence of double-positive CD4+CD8+ thymocytes and of peripheral T cells [30]. RAFTLIN (raft-linking protein) was also shown to influence T-cell mediated immune responses and Th17 differentiation [31]. STK17B is a serine kinase also known as DRAK (DAP kinase-related apoptosis-inducing kinase) 2 which is known to mediate apoptosis induced by IL-2 and regulate T cell receptor sensitivity in developing thymocytes [32]–[33]. Phosphodiesterase (PDE) 4 B is a type 4 PDE (up ∼2-fold) that regulates TCR signaling by tempering the negative effect of cAMP [34] and in CD4+ human T cells decreased PDE4B expression leads to reduced IL-2 production upon anti-CD3/CD28 co-stimulation [35]. The chip-on-ChIP identified STAT5 binding site within the PDE4B is shown in Figure 4B, visualized by the IGV. Intriguingly, IL-2 increased the level of PDE4B, which contains several intronic STAT5 binding sites, based on murine T cell studies [5].

**Table 1:** Validation of STAT5-dependent, IL-2-mediated gene expression changes.

			Fold Response (to ctrl)		p-value (to ctrl ) <0,05		
	GL	Symbol	3 h	6 h	3 h	6 h	
1	En	**AHR**	4.53	**4.41**	0.0722	**0.0177**	
2	Ex	**BCL2**	**2.75**	2.07	**0.0500**	0.1099	*
3	Pp	*BCL6*	***−3.76***	***−4.19***	***0.0001***	***0.0207***	*
4	Pp	**CD69**	**2.29**	**2.28**	**0.0500**	**0.0500**	
5	ID	**CDK6**	**3.61**	**3.34**	**0.0091**	**0.0089**	*
6	En	*CDKN2C*	−1.80	***−1.96***	0.0674	***0.0312***	
7		**CISH**	**28.73**	**21.18**	**0.0001**	**0.0002**	*
8	Pp	**EMP1**	**3.06**	1.69	**0.0496**	0.3392	
9	Pp	*EPHA4*	***−8.67***	***−14.95***	***0.0051***	***0.0002***	
10		**FOXP3**	**4.04**	**3.03**	**0.0035**	**0.0090**	*
11	Pp	**IDI1**	**2.09**	**2.01**	**0.0209**	**0.0379**	
12		**IFNG**	**15.19**	**11.19**	**0.0086**	**0.0403**	*
13	Pp	**IL2RA**	**7.44**	**6.22**	**0.0010**	**0.0015**	*
14	Pp	**LCP2**	**1.79**	**1.66**	**0.0384**	**0.0078**	
15	ID	*LRMP*	***−3.12***	−25.91	***0.0044***	0.2041	
16	ID	**MKI67IP**	**1.60**	**1.73**	**0.0153**	**0.0064**	
17	Ex	**MMD**	**2.43**	**2.14**	**0.0092**	**0.0512**	
18	Pp	**NETO2**	**2.87**	**2.58**	**0.0369**	**0.0210**	
19	Pp	**PDE4B**	**2.19**	**1.96**	**0.0042**	**0.0058**	
20	En	*RAFTLIN*	1.01	***−1.36***	0.9631	***0.0229***	
21	Ex	**SLC2A3**	**1.75**	**1.63**	**0.0075**	**0.0215**	
22	Pp	**STIP1**	**3.25**	**2.55**	**0.0010**	**0.0309**	
23	En	**STK17B**	1.49	**1.44**	0.0994	**0.0185**	
24	Pp	**UBE2W**	**2.35**	2.44	**0.0500**	0.0639	
25	En	**UGP2**	1.57	**1.55**	0.1079	**0.0446**	
26	En	**WDR3**	1.73	**1.56**	0.1103	**0.0416**	

Transcript level changes of 57 intersect genes were measured using qRT^2^PCR arrays in PHA activated, quiescent and IL-2 stimulated (3 and 6 h) PBMCs (72 h activated, 48 h quiescent, from 3 independent donors). Fold response and the p-value to the un-stimulated control samples are shown. Bold letters indicate significantly up-regulated, while italic letters represent significantly down-regulated genes. Stars specify known target genes. Genomic locations are marked as follows: Pp, Proximal Promoter; ID, Immediate Downstream; En, Enhancer; E, Exon. Unlabeled boxes contain genes that were identified by GEA but not chip-on-ChIP.

### STAT5 Occupies a Putative IL-2 Responsive GAS Site within the PDE4B Gene *in vivo* in Human PBMCs

**Figure 5:**
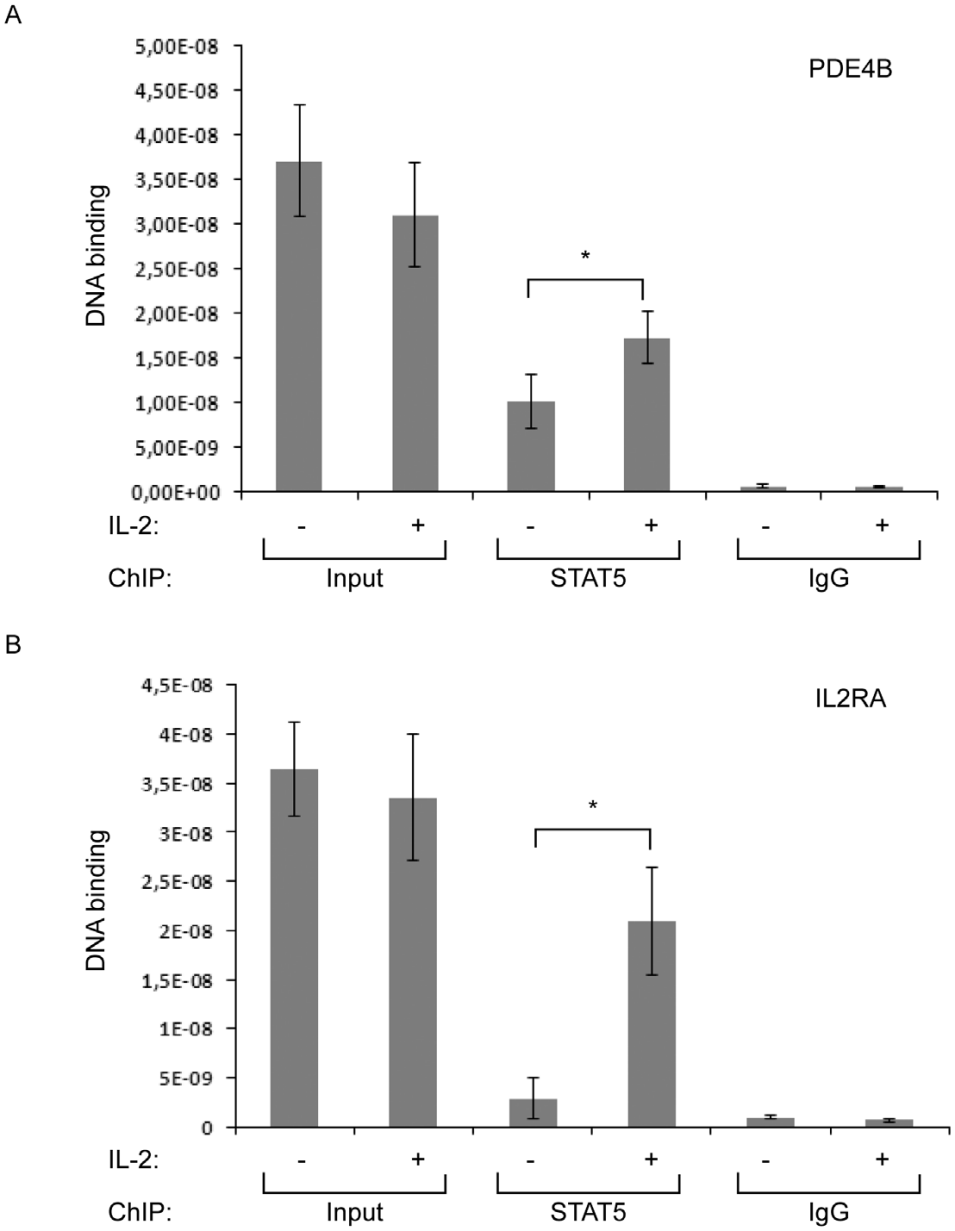
STAT5 binds PDE4B in an IL-2 inducible manner in hPBMC. ChIP assays performed with STAT5 antibodies or control sera (IgG) were carried out in quiescent (−) or IL-2 stimulated (30 min, +) hPBMCs isolated from three independent donors to measure the enrichment of the PDE4B putative STAT5 regulated region (**A**) or the IL2RA enhancer PRR III (**B**) identified by chip-on-ChIP. Inputs represent 1% of chromatin used in ChIP assays and error bars represent standard deviations. * represents statistically significant differences (p<0.05).

To confirm that STAT5 is able to bind a putative GAS site within the PDE4B gene (located on chromosome 1, hg18 positions 66567898–66573077) in an IL-2 inducible manner, we performed ChIP experiments in quiescent human PBMCs left untreated (−) or treated with IL-2 for 30 min (+) isolated from three independent donors. As a result we observed that STAT5 bound PDE4B in an IL-2 dependent and significant manner (p<0.05) with an approximately 1.7-fold increase as compared to the untreated cells (Figure 5A). The IL2RA PRR III was used as a positive control (Figure 5B). Interestingly, the PDE4B gene contained both STAT5A and STAT5B IL-2 responsive binding sites investigated within primary human T cells [16] that overlapped with our chip-on-ChIP region. Moreover, the increased STAT5 DNA binding activity following IL-2 treatment correlated well with the observed 2-fold increase in mRNA levels (Table 1).

### Short Term IL-2 Treatment (3 h) Induces PDE4B Protein Expression in PHA-activated Human PBMCs and CD8+ but not CD4+ Cells

**Figure 6:**
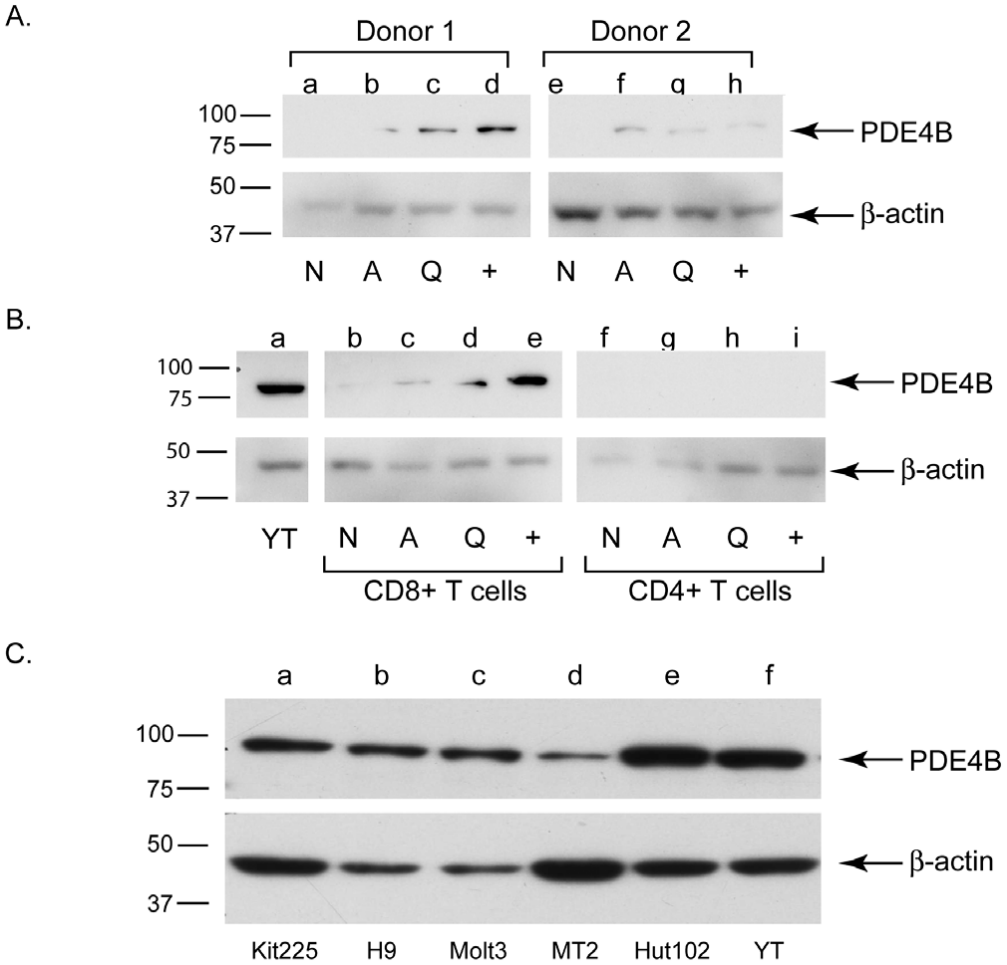
IL-2 stimulation induces PDE4B in activated human PBMCs (A) and CD8+ but not CD4+ T-cells (B). Normal human PBMCs from three independent donors (shown are 2 representative results) were left un-activated (N, naïve) or were activated with PHA (A, Q, +) then made quiescent after 72 hrs PHA activation by CO_2_ stripping as described in the Materials and Methods section (Q), then stimulated with IL-2 for 3 h (+). Cells were harvested and then Western blotted with antibodies to PDE4B or ß-actin as indicated to the right. Molecular weight markers are shown to the left. In the lower panel immediately after PBMC isolation CD4+ (lanes f-i) or CD8+ (lanes b-e) cells were negatively selected as described in the Materials and Methods section using magnetic beads, and then activated with PHA, made quiescent, stimulated with IL-2 and Western blotted for PDE4B and ß-actin as described above. YT cells served as positive controls for PDE4B expression. (**C**) **PDE4B is expressed in CD4+ lymphoid tumor cell lines.** Kit225, H9, Molt3, MT2, Hut102 CD4+ and YT NK-like lymphoid cell lines were lysed and equal amounts of protein Western blotted for PDE4B and ß-actin as described for Figure 6A. Representative data from two independent experiments is presented.

To further validate that PDE4B is regulated by IL-2, we also examined protein level changes in naïve (N), PHA-activated (A), quiescent (Q) and IL-2 stimulated human PBMCs at 3 h (+) in two independent donors (Figure 6A). The level of PDE4B protein increased approximately 2-fold (Figure S4, densitometry analysis), which correlates with the ∼1.7-fold increase in DNA binding activity of STAT5 and 2-fold increase in mRNA levels (Figure 5A and Table 1, respectively). Primary human CD4+ or CD8+ T-cells were also tested (Figure 6B). YT NK-like cells served as positive control and ß-actin as a loading control. Both PHA-activation (72 h) and short term IL-2 treatment induced PDE4B protein expression in PBMCs and CD8+ but not CD4+ T-cells. Based on this data it is interesting to speculate that PDE4B might be the first “line of defense” in PBMCs and CD8+ T-cells against the elevated cAMP signaling that occurs with T-cell stimulation [36]. It is known that another type 4 isoform, PDE4D is also expressed in CD4+ T-cells [35], which leads us to speculate a possible model where there is constant tempering of cAMP induced pathways as compared to CD8+ cells. Another possibility might be that the delayed up-regulation of PDE4B in CD4+ cells is to down-regulate cAMP in these cells at a distal point of time. Testing of CD4+ vs CD8+ cells was performed from a single donor and we believe provides new opportunities to investigate the possible role of PDE4B in these types of T cell subsets.

### PDE4B is Expressed in CD4+ Lymphoid Tumor Cell Lines

Since PDE4B was found abnormally expressed in diffuse large B-cell lymphoma samples [35] the question arose what could be the status of PDE4B expression in various CD4+ and other lymphoid cancer cell lines. Therefore, a set of human CD4+ lymphoid tumor cell lines and an NK-like cell line, YT, were examined by Western blotting that showed highly expressed PDE4B protein present in these cells (Figure 6C). STAT5 signaling pathway is relevant to several of these cell lines: human MT2 and Hut102 and display hyperactive STAT5 pathway [37], while YT and Kit225 cells undergo apoptosis when STAT5 is depleted [11], [38]. Intriguingly, PDE4B was found over-expressed in the fraction of diffuse large B-cell lymphoma samples that were refractory to chemotherapy [39]. It would be therefore interesting to investigate cell fate if PDE4B could be effectively depleted and whether death may result.

Taken together, it is plausible to postulate that overexpression of PDE4B might be the result of abnormal genomic DNA binding of STAT5 that could subsequently contribute to the tumorigenic phenotype of these cell lines. Testing of this hypothesis requires further investigation.

### Conclusions

In conclusion, utilizing a systematic approach that combined the determination of the IL-2 induced STAT5 cistrome and gene expression analysis within a human leukemia cell model; and with follow-up gene ontology as well as medium throughput transcript expression validation in primary human PBMCs we have identified 19 novel STAT5 target genes, some with functions yet-to-be determined and some with relevance to immune cells. We also validated a novel candidate target gene, PDE4B at the protein level in PBMCs and found it over-expressed in CD4+ lymphoid tumor lines. These data also suggests that tumor cells of lymphoid origin might have skewed genomic STAT5 binding sites and hence target genes as compared to normal lymphoid cells (as suggested by the comparison of our data with already existing data in the literature by Liao and colleagues [16]); therefore, finding specific targets to eradicate them might require genome-wide mapping of larger sets of primary tumor samples of the same origin. Whether lymphoid cells with an overactive STAT5 pathway might be sensitive to PDE4B inhibitors and a mechanism to control certain tumors will be the subject of future studies.

## Materials and Methods

### Cell Culture and Treatment

The human lymphoma cell line YT [40], CD4+ human T-cell lines Hut-102 [41] and MT-2 [42], thymus-derived CD4+ T-lymphocyte cell line Molt-3 [43], CD4+ T-cell line H9 [44] and human CD4+ IL-2 dependent leukemia cell line Kit225 [45] (kindly provided by Dr. J. Johnston, Queens University, UK) were cultured as described [4], [38]. IL-2 was obtained from the NCI Preclinical Repository. Human peripheral blood mononuclear cells (PBMC) were isolated, activated with PHA and maintained as described [46]. CD4+ or CD8+ T-cells were isolated by negative selection using Dynabeads® Untouched™ Human CD4 T Cells (Cat. no. 113.46D) or Human CD8 T Cells (Cat. no. 113.48D) kits, respectively.

### Chromatin Immunoprecipitation

Chromatin immunoprecipitations were performed from approximately 5×10^7^ Kit225 cells as described [4] with anti-STAT5A/B antibodies (mixing sc-1081 and sc-835 (C-terminal STAT5A and STAT5B antibodies, respectively)) or normal rabbit serum (IgG control) for 3 h at 4°C. To confirm that the assays were successful, PCR amplification of the known STAT5 binding site located 5′ to the human IL2RA gene within the Positive Regulatory Region III [23] (Forward: 5′-ACG TCT AGA AAG AAA GTG GTC-3′ Reverse: 5′- CTG TCC CTG GAT GAA CCT AGT-3′) was performed using quantitative real time PCR. [4] Values of transcripts in unknown samples were obtained by interpolating Ct (PCR cycles to threshold) values on a standard curve. Standard curves were prepared from known amounts of purified, PCR-amplified DNA. ChIP qPCR primer assays for PDE4B ChIP validation were ordered from SABiosciences, a Qiagen company (Cat# GPH900044(−)01A), by providing chromosomal positions for the 250 bp surrounding the putative GAS site (chr1∶66569949–66570198, hg18). PCR reactions were carried out using 2× SYBR Green Mastermix from BioRad and a BioRad iQ5 thermocycler in triplicates. Arbitrary units defined as “DNA binding, (Db)” was obtained by 2∧(−averageCt) and standard deviation as SD ≈ ln(2) * stdev(averageCt) * Db.

### Random Amplification of Chromatin Immunoprecipitated DNA

To generate libraries from ChIP-ed DNA, random amplification was carried out as described at http://research.stowers-institute.org/gertonlab/protocols/RandomPCRamplification.pdf by the DeRisi lab at the UC San Francisco. Briefly, ChIP-ed DNA samples were amplified with a modified version of a random PCR protocol [47]. DNA was annealed to primer A (5′-GTTTCCCAGTAGGTCTCNNNNNNNN), and second-strand DNA synthesis was carried out with Sequenase (US Biochemical). This material was then used as the template for 35 cycles of PCR with Primer B (5′-GTTTCCCAGTAGGTCTC) using the following profile: 30 s at 94°C, 30 s at 40°C, 30 s at 50°C, 60 s at 72°C and a dNTP mix containing dUTP. The products were purified with QIAGEN PCR purification kit, quantified and run on a 1% agarose gel to check for fragment size, then tested for enrichment of the Positive Regulatory Region III as described above.

### *In Silico* Analysis

Chip-on-ChIP results were analyzed with MAT (Model-based Analysis of Tiling array, [24] http://liulab.dfci.harvard.edu/MAT/) by the Liu Lab in the Department of Biostatistics and Computational Biology at the Dana-Farber Cancer Institute, Harvard School of Public Health. Proximal gene mapping of the genomic sequences up to 300 kb was performed using the Cis-Regulatory Element Annotation System (CEAS http://liulab.dfci.harvard.edu/CEAS/). The results were visualized by the IGV. The conversion of genome coordinates and genome annotation files of different versions of the human genome assemblies was performed using UCSC Genome Liftover tool at http://genome.ucsc.edu/cgi-bin/hgLiftOver.

### RNA Isolation and cDNA Synthesis

Total RNA was isolated using the RNeasy kit (QIAGEN). cDNA was synthesized with BioRad’s iScript cDNA Synthesis Kit according to the manufacturer’s instructions.

### Microarray Analysis

Gene Expression Analysis using Affymetrix Human Genome U133 Plus 2.0 microarrays were carried out at the Microarray Core Facility, Baylor College of Medicine, Houston, TX. Statistical analysis was performed using GeneSpring GX at the University of Debrecen. Affymetrix GeneChip Human Promoter 1.0R arrays interrogate regions proximal to transcription start sites and contain probes for approximately 59 percent of CpG islands. These arrays contain 4.6 million probes tiled through over 25,500 human promoter regions at an average resolution of 35 bp. Each promoter region covers approximately 7.5 kb upstream through 2.45 kb downstream of 5′ transcription start sites. For over 1,300 cancer-associated genes, coverage of promoter regions was expanded to include additional genomic content. Fragmentation and microarray hybridization to Affymetrix GeneChip Human Promoter 1.0R arrays were carried out according to the manufacturer’s instructions by the Genomics Core Facility at the European Molecular Biology Laboratory in Heidelberg, Germany. The data is available at the Gene Expression Omnibus Database (https://www.ncbi.nlm.nih.gov/projects/geo/Accession: GSE40624).

### Gene Ontology Analysis

To identify overrepresented GO categories within the IL-2 regulated genes, the Ingenuity Pathway Analysis software was used.

### Cell lysis and Western Blotting

Cell lysis and Western blots were performed as previously described [38] with antibodies to PDE4B (Abnova, Catalog #: PAB6965) and ß-actin (Sigma-Aldrich). The antibodies were used at a dilution recommended by the manufacturer. Densitometry analysis was performed using the Un-Scan-It version 6.1 software by Silk Scientific, Inc.

### RT^2^ Profiler PCR Arrays

To determine the expression profile of genes regulated by IL-2, SA Biosciences’ Human PCR Arrays were used. Quantification based on real-time monitoring of amplification was determined using a BioRad iQ5 thermocycler and 2× SYBR Green Mastermix (SA Biosciences, a Qiagen company). Treatments were performed in triplicates and data was analyzed using the ΔΔC_t_ method.

### Statistical Analysis

Normalized t-tests were performed using SigmaStat 3.1.

## Supporting Information

Figure S1 **Experimental design of a genome-wide approach to identify STAT5 specific IL-2-induced genes.** Kit225 IL-2-dependent human leukemia cells were stimulated with IL-2, cross-linked with formaldehyde then chromatin immunoprecipitated with antibodies to STAT5A/B. Eluted DNA was amplified then probed against human Affymetrix Promoter arrays and data analyzed to generate a pool of genomic locations with putative and known STAT5 binding sites. (Flow chart on the left.) Quiescent Kit225 cells were left un-stimulated or were stimulated with IL-2 for 3 hours then Gene Expression Analysis (GEA) performed to detect IL-2 responsive genes. (Flow chart on the right.) The data pools then were aligned using UCSC Genome Browser then gene expression changes of select genes from the overlapping hits were validated using SABiosciences qRT^2^PCR arrays in PHA activated quiescent PBMCs isolated from three independent donors.
(TIF)

Figure S2 **Pathway analysis predicted activation of STAT5A and STAT5B transcription factors by IL-2.** Based on the appearance of their target genes in the IL-2 regulated gene list (GEA analysis, 469 genes changed, 340 up- and 129 genes down-regulated) Ingenuity Pathway Analysis created the network of genes visualized by their subcellular localization. Red indicates up- and green shows down-regulated genes.
(TIF)

Figure S3 **IL-2 regulated top networks based on Ingenuity Pathway Analysis of GEA results.** Cellular Development & Cell Cycle, Hematological System Development & Function, Reproductive System Development & Function as well as Cellular Movement, Growth & Proliferation were found significantly overrepresented within the GEA generated gene list.
(TIF)

Figure S4 **Densitometry analysis of PDE4B protein expression in hPBMC.** Arbitrary units were generated by the Un-Scan-It v6.1 software counting total pixels of the bands in the Western blot images in Fig6A for both ß-actin and PDE4B in both donors, and then the ratio of PDE4B/ß-actin was generated and compared to the naïve (N) samples. Error bars represent standard deviations.
(TIF)

Table S1 **Select putative IL-2 sensitive STAT5 target genes.** Table shows the 57 intersect genes chosen for validation with their genomic locations in relation to the corresponding regulated gene as well as their RefSeq IDs. Genomic locations are marked as follows: Pp, Proximal Promoter; ID, Immediate Downstream; En, Enhancer; E, Exon.
(XLSX)
