# Correction: Genome Wide Mapping Reveals PDE4B as an IL-2 Induced STAT5 Target Gene in Activated Human PBMCs and Lymphoid Cancer Cells

**DOI:** 10.1371/annotation/cb55d096-61f8-46de-b58f-81a872be6dd3

**Published:** 2013-02-27

**Authors:** Zsuzsanna S. Nagy, Jeremy A. Ross, Georgialina Rodriguez, Balint L. Balint, Lajos Szeles, Laszlo Nagy, Robert A. Kirken

There is an error in the Funding statement. The correct Funding statement is:

This work was supported by the National Institutes of Health (NIH) grant number AI053566, Lizanell and Colbert Coldwell Foundation, Edward N. and Margaret G. Marsh Foundation, and Grant G12MD007592 from the National Institutes on Minority Health and Health Disparities (NIMHD), a component of the National Institutes of Health (NIH) to RAK and a pilot project grant to Z.S.N. (NIGMS SCORE program, grant S06 GM008012-37). Z.S.N. is the recipient of the János Bolyai Research Fellowship from the Hungarian Academy of Sciences and is supported by the NKTH-OTKA-EU 7KP (Marie Curie actions) Reintegration Grant (OTKA MB08C 84630). The funders had no role in study design, data collection and analysis, decision to publish, or preparation of the manuscript. 

