# Supplementary figures and images for: Genome Wide Mapping Reveals PDE4B as an IL-2 Induced STAT5 Target Gene in Activated Human PBMCs and Lymphoid Cancer Cells
Source: PLoS One. 2013 Feb 25;8(2):e57326. doi: 10.1371/journal.pone.0057326 (PMC3581501; doi:10.1371/journal.pone.0057326)

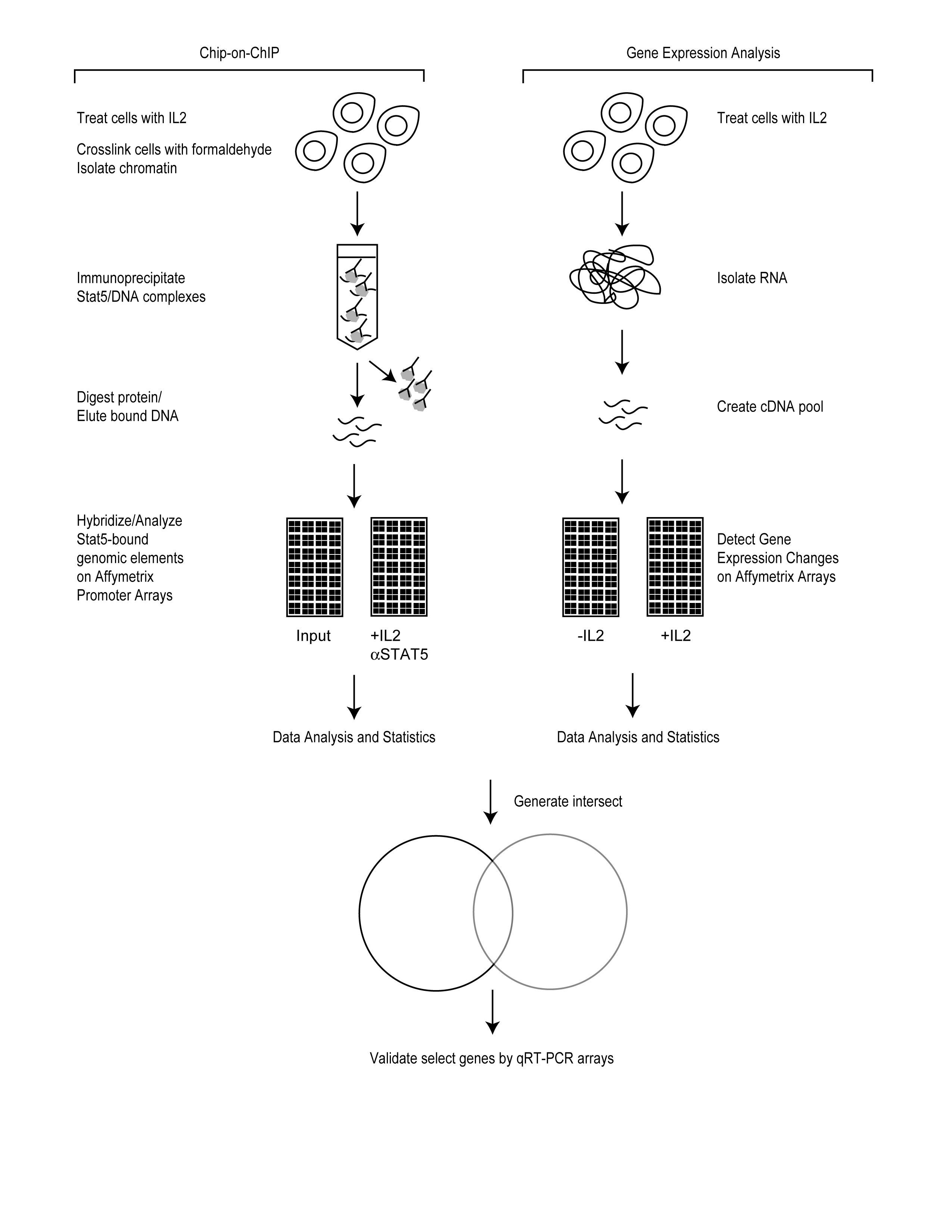

Supplement: Figure S1 — Experimental design of a genome-wide approach to identify STAT5 specific IL-2-induced genes. Kit225 IL-2-dependent human leukemia cells were stimulated with IL-2, cross-linked with formaldehyde then chromatin immunoprecipitated with antibodies to STAT5A/B. Eluted DNA was amplified then probed against human Affymetrix Promoter arrays and data analyzed to generate a pool of genomic locations with putative and known STAT5 binding sites. (Flow chart on the left.) Quiescent Kit225 cells were left un-stimulated or were stimulated with IL-2 for 3 hours then Gene Expression Analysis (GEA) performed to detect IL-2 responsive genes. (Flow chart on the right.) The data pools then were aligned using UCSC Genome Browser then gene expression changes of select genes from the overlapping hits were validated using SABiosciences qRT2PCR arrays in PHA activated quiescent PBMCs isolated from three independent donors. (TIF) [file pone.0057326.s001.tif]

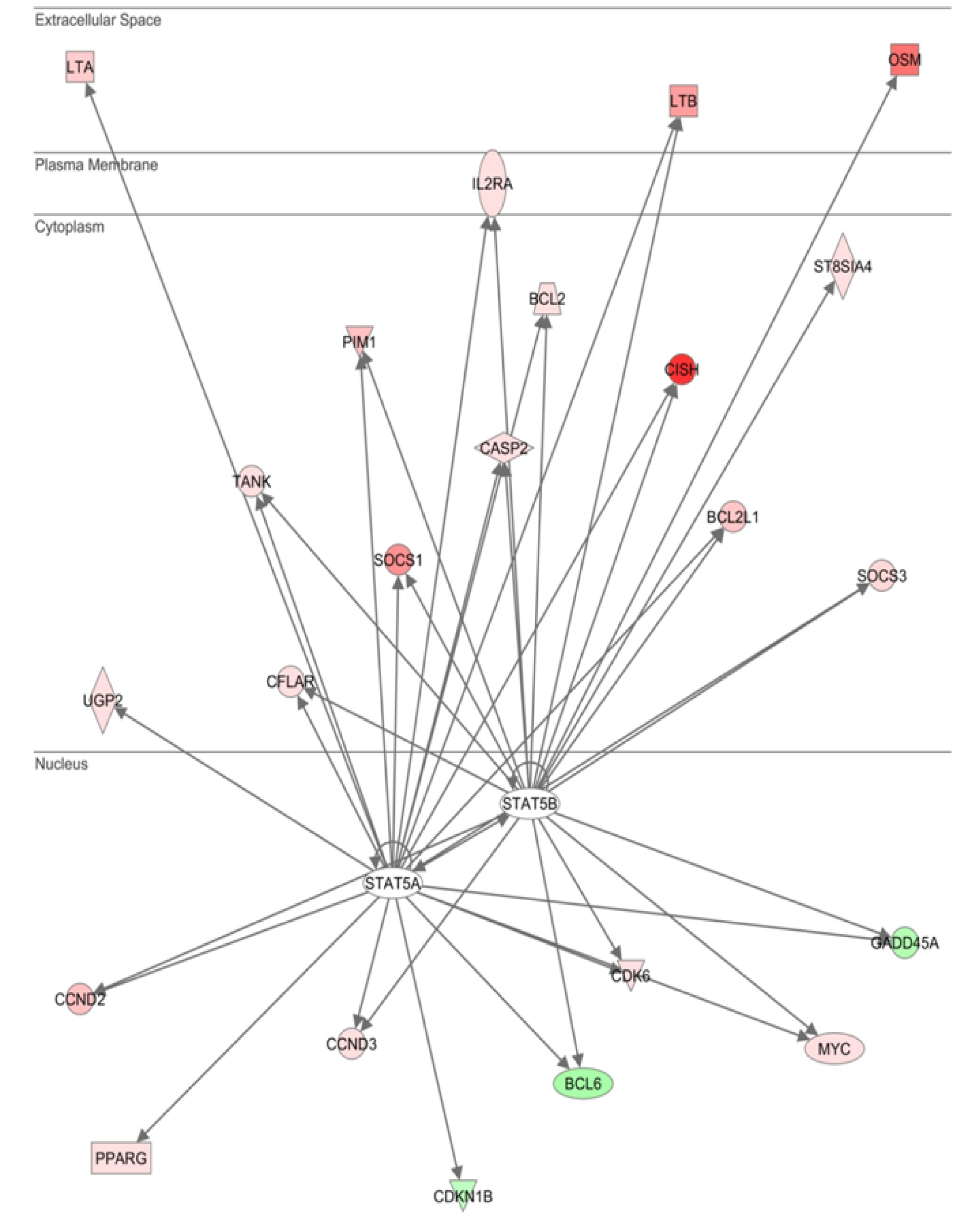

Supplement: Figure S2 — Pathway analysis predicted activation of STAT5A and STAT5B transcription factors by IL-2. Based on the appearance of their target genes in the IL-2 regulated gene list (GEA analysis, 469 genes changed, 340 up- and 129 genes down-regulated) Ingenuity Pathway Analysis created the network of genes visualized by their subcellular localization. Red indicates up- and green shows down-regulated genes. (TIF) [file pone.0057326.s002.tif]

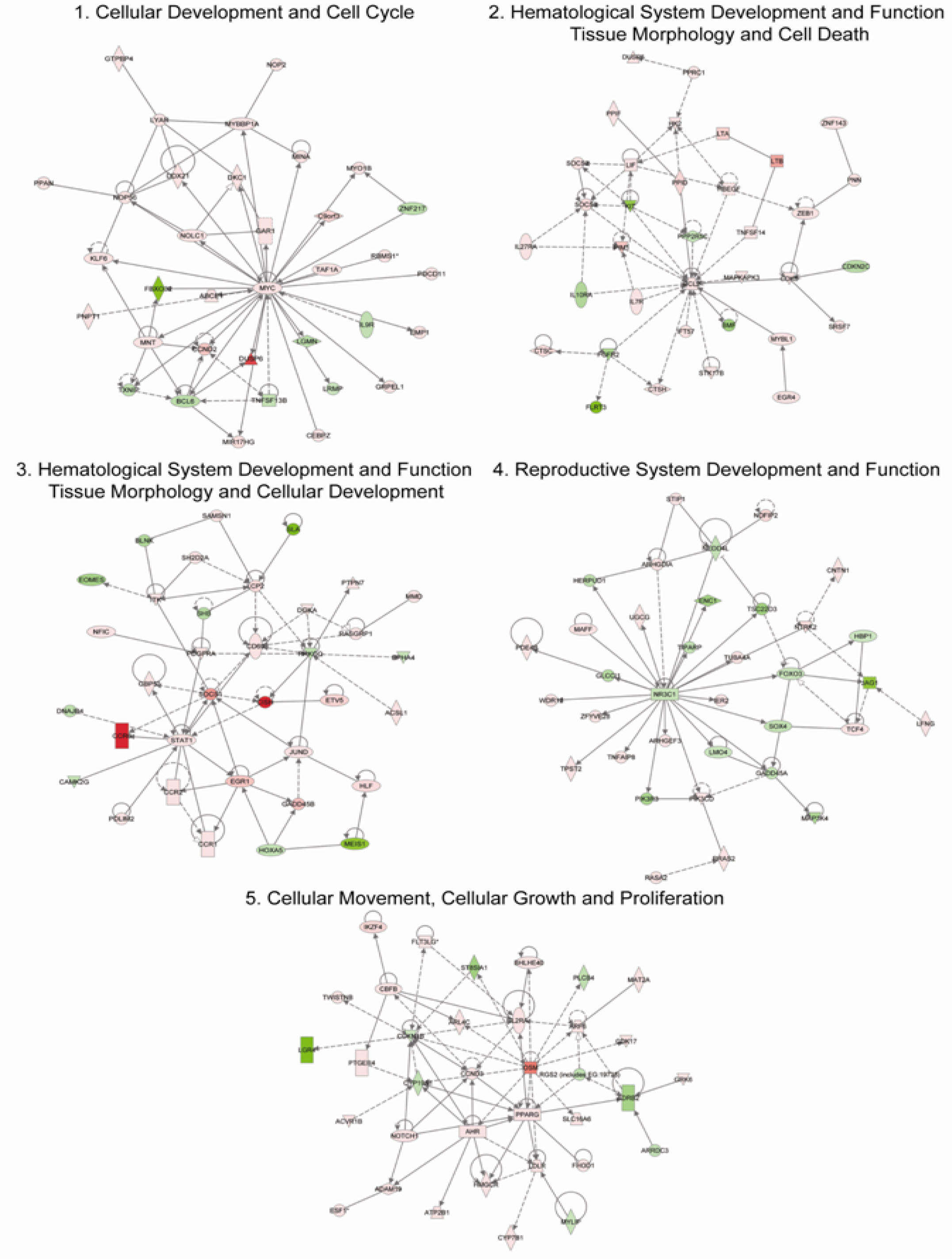

Supplement: Figure S3 — IL-2 regulated top networks based on Ingenuity Pathway Analysis of GEA results. Cellular Development & Cell Cycle, Hematological System Development & Function, Reproductive System Development & Function as well as Cellular Movement, Growth & Proliferation were found significantly overrepresented within the GEA generated gene list. (TIF) [file pone.0057326.s003.tif]

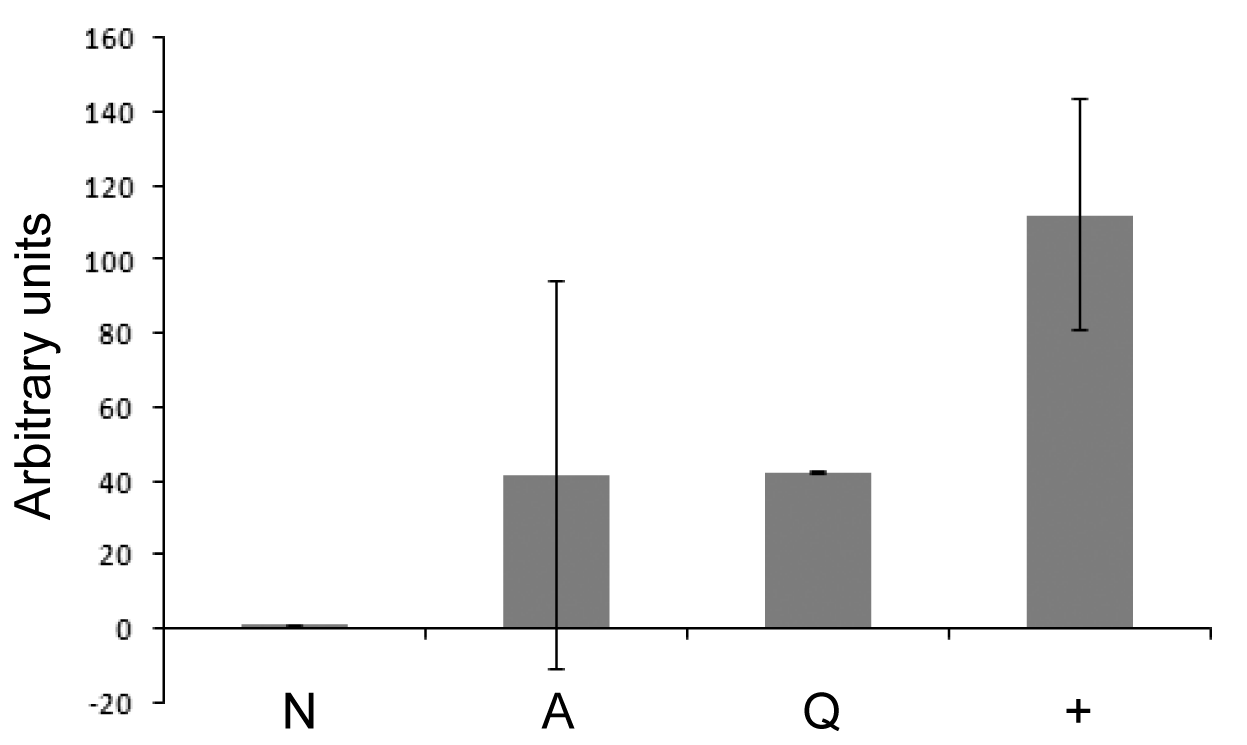

Supplement: Figure S4 — Densitometry analysis of PDE4B protein expression in hPBMC. Arbitrary units were generated by the Un-Scan-It v6.1 software counting total pixels of the bands in the Western blot images in Fig6A for both ß-actin and PDE4B in both donors, and then the ratio of PDE4B/ß-actin was generated and compared to the naïve (N) samples. Error bars represent standard deviations. (TIF) [file pone.0057326.s004.tif]
